# Estimating species richness using environmental DNA

**DOI:** 10.1002/ece3.2186

**Published:** 2016-05-30

**Authors:** Brett P. Olds, Christopher L. Jerde, Mark A. Renshaw, Yiyuan Li, Nathan T. Evans, Cameron R. Turner, Kristy Deiner, Andrew R. Mahon, Michael A. Brueseke, Patrick D. Shirey, Michael E. Pfrender, David M. Lodge, Gary A. Lamberti

**Affiliations:** ^1^ Department of Biological SciencesUniversity of Notre DameNotre DameIndiana; ^2^ Shrimp DepartmentOceanic Institute at Hawai'i Pacific UniversityWaimanaloHawaii; ^3^ Biology DepartmentUniversity of NevadaRenoNevada; ^4^ Department of BiologyInstitute for Great Lakes ResearchCentral Michigan UniversityMount PleasantMichigan

**Keywords:** Chao estimator, electrofishing, freshwater community, metabarcoding, species identity

## Abstract

The foundation for any ecological study and for the effective management of biodiversity in natural systems requires knowing what species are present in an ecosystem. We assessed fish communities in a stream using two methods, depletion‐based electrofishing and environmental DNA metabarcoding (eDNA) from water samples, to test the hypothesis that eDNA provides an alternative means of determining species richness and species identities for a natural ecosystem. In a northern Indiana stream, electrofishing yielded a direct estimate of 12 species and a mean estimated richness (Chao II estimator) of 16.6 species with a 95% confidence interval from 12.8 to 42.2. eDNA sampling detected an additional four species, congruent with the mean Chao II estimate from electrofishing. This increased detection rate for fish species between methods suggests that eDNA sampling can enhance estimation of fish fauna in flowing waters while having minimal sampling impacts on fish and their habitat. Modern genetic approaches therefore have the potential to transform our ability to build a more complete list of species for ecological investigations and inform management of aquatic ecosystems.

## Introduction

The foundation for ecological insights into species interactions, food web dynamics, and ecosystem functioning is accurately measuring species number and identity (Mace et al. 2012). Biodiversity is a key element of ecosystem function (Risser 1995) and species richness is a fundamental measure of that diversity, underlying many ecological concepts and models (Gotelli and Colwell 2011). A fundamental challenge to increasing accuracy of biodiversity estimates is to improve detection probabilities for uncommon, rare, or elusive species (Gu and Swihart 2004). For conservation management, the presence of a rare species can help delimit critical habitat necessary to protect threatened and endangered species (Arponen et al. 2005), or identify new invasive species that may fundamentally shift community structure (Didham et al. 2007). Ultimately, current technology and methods must improve to better identify invasive, threatened, or endangered species or to document biodiversity, a critical metric for assessing environmental change (Butchart et al. 2010).

Conservation biologists use three approaches to improve species detection rates or to infer richness. The first approach is to increase effort. If a species has some nonzero probability of detection, then theory indicates additional sampling will eventually detect all species, including the rarest (McDonald 2004). An indefinite search is generally not practical, however, with limited resources such as time and money. A second approach is to estimate the number of unobserved species from samples of observed species (Chao et al. 2009). These estimators use information about the frequency of a species' occurrence in only one and two samples to predict the number of species that may be undetected. The identities of the undetected, but inferred species are, however, unknown. The third approach is to change detection methods (McDonald 2004). Technological advances have significantly improved our ability to estimate species richness by providing new methods such as remote sensing platforms capable of reading light signatures to allow detection of different plant species (Turner et al. 2003). Another emerging technique is the use of environmental DNA (eDNA) and high‐throughput sequencing to detect species in aquatic environments (Thomsen et al. 2012). Species leave genetic signatures in the environments they inhabit, which can be analyzed to increase species detection probabilities (Mahon et al. 2014; Evans et al. 2015; Simmons et al. 2015; Valentini et al. 2015).

Fish communities are a particularly suitable taxonomic group to explore the utility of an eDNA metabarcoding method to increase detection rates because they often have low local diversity and are generally well sampled for specific water bodies or watersheds (Lodge et al. 2012). Additionally, small streams (i.e., wadeable) are quite tractable for fish inventories because discrete areas can be effectively delineated and sampled. Fish are, however, mobile species in watersheds and typically move in and out of local patches across a wide range of habitats within a watershed. Therefore, a fish species often requires knowing the specific timing or location where it is found in order to capture it. However, eDNA shed into the water column may persist after a species has moved through a habitat patch or reach of a stream or river (Fukumoto et al. 2015). Consequently, lotic ecosystems (i.e., streams and rivers) represent an amalgamation of eDNA from many geographic sources (Thomsen and Willerslev 2015) and provide an opportunity to sample numerous species independent of their local and potentially patchy distribution, reducing the sampling effort required by more traditional methods (Deiner et al. 2015).

In this study, we utilize a historically well‐characterized fish fauna from a northern Indiana, USA, stream that had 17 continuous years of sampling via depletion‐based electrofishing. Here, we evaluate the efficacy of eDNA relative to electrofishing to test whether the use of eDNA can increase detection efficiency, maintain accuracy when claiming a positive detection using genetic fragments, and increase identification of species in a community.

## Materials and Methods

### Study site

**Figure 1:**
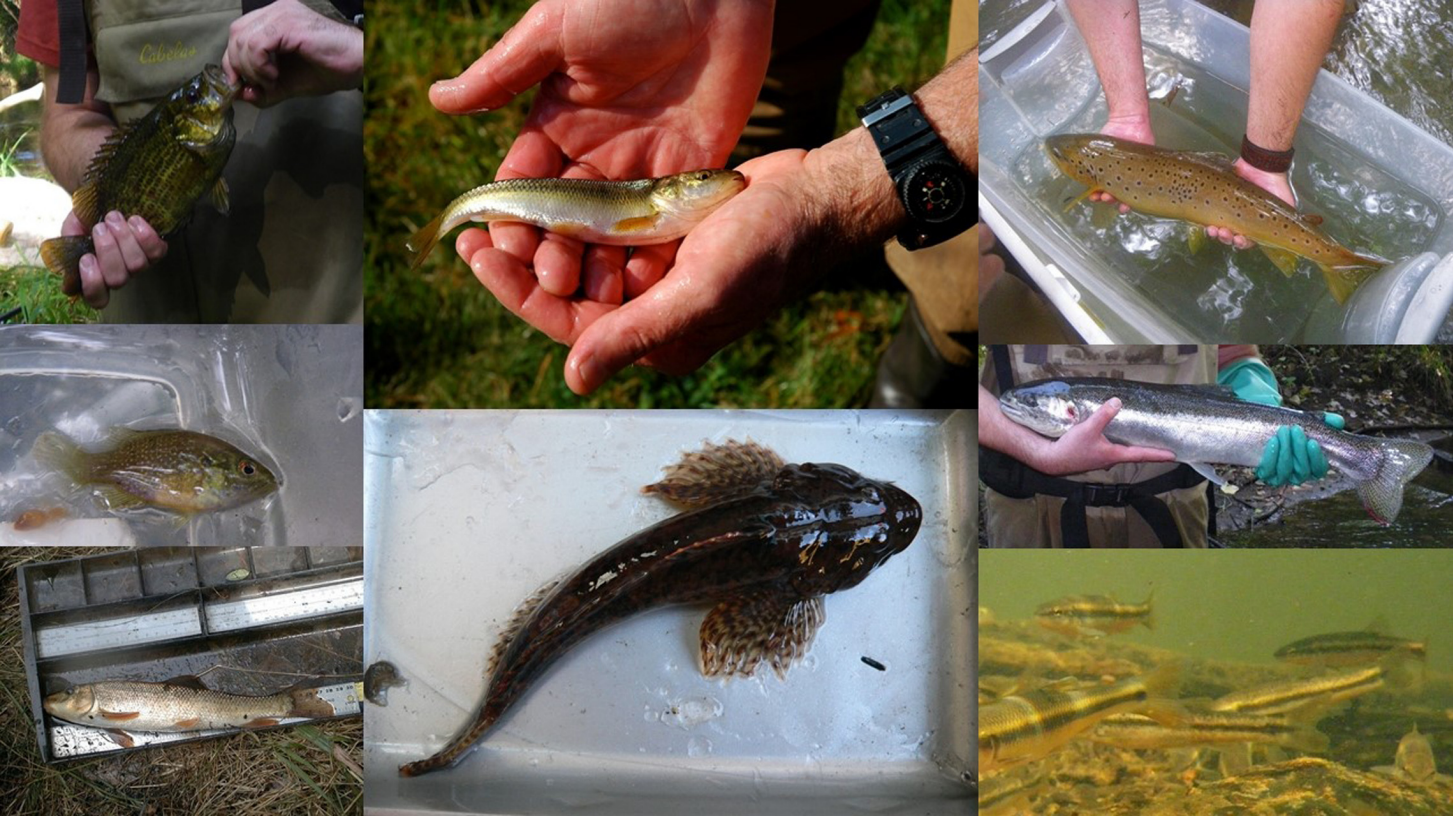
Sample of fish species inhabiting Juday Creek (clockwise from top left; Rock bass, Creek chub, Brown trout, Steelhead, Blacknose dace, Mottled sculpin, White sucker, and Green sunfish).

Juday Creek is a small third order tributary (1997–2013 mean annual discharge = 0.44 m^3^/sec) to the St. Joseph River in northwestern Indiana, USA. As part of a long‐term study of a 1200‐m‐long stream section that flows through the University of Notre Dame campus, four discontinuous 60‐m reaches (each separated by ~100 m) were established in 1997 and have been sampled annually via backpack electrofishing conducted by the University of Notre Dame Stream and Wetland Ecology Laboratory. This intensive annual sampling has produced a list of 18 known fish species in the system (Table 1; Fig. 1). Across this time span, we observed many species only a few times, while others were observed every year.

**Table 1:** Historical record of species captured for all reaches within Juday Creek from 1997–2013

Common name	Scientific name	No. of years captured (of 17 total years)	Last year collected
Creek chub	*Semotilus atromaculatus*	17	2013
Green sunfish	*Lepomis cyanellus*	17	2013
Johnny darter	*Etheostoma nigrum*	17	2013
Mottled sculpin	*Cottus bairdii*	17	2013
Western blacknose dace	*Rhinichthys obtusus*	17	2013
White sucker	*Catostomus commersonii*	17	2013
Rainbow trout	*Oncorhynchus mykiss*	15	2013
Brown trout	*Salmo trutta*	13	2013
Rock bass	*Ambloplites rupestris*	11	2013
Smallmouth bass	*Micropterus dolomieu*	11	2013
Bluegill sunfish	*Lepomis macrochirus*	10	2013
Largemouth bass	*Micropterus salmoides*	6	2012
Pumpkinseed sunfish	*Lepomis gibbosus*	4	2010
Yellow perch	*Perca flavescens*	3	2005
Central mudminnow	*Umbra limi*	2	2011
Rainbow darter	*Etheostoma caeruleum*	2	2013
Golden shiner	*Notemigonus crysoleucas*	1	2008
Warmouth	*Lepomis gulosus*	1	2012

### Electrofishing sampling

In 2013, we assessed the Juday Creek fish assemblage as carried out in previous years in each of the four 60‐m study reaches using block‐netted, sequential depletion, triple‐pass, pulsed DC electrofishing using a Model 12, 12‐B, or LR‐24 backpack electrofisher (Smith‐Root Inc., Vancouver, WA). Typically, one person operated the electrofisher while three to five people dip‐netted. We identified captured fishes to species prior to releasing them alive back into the stream (Moerke and Lamberti 2003). Annual sampling was generally conducted in early fall, and in 2013, we performed electrofishing of the four reaches on September 11 and 13.

### eDNA sampling

Sampling for eDNA was also conducted on 11 and 13 September 2013, just prior to annual electrofishing, in two reaches each day, for a total of four reaches. Before electrofishing, we collected two 250‐mL water samples from each reach, one each at the downstream and upstream boundary, for eight total samples. We sampled at the most downstream location first and then moved upstream within and between reaches. To avoid disturbing sediment or organisms, we used an extended pole with a dip bucket to collect surface water from the bank. The pole and dip bucket were wiped with 10% bleach and rinsed with RO water before each collection. For each reach (*n* = 4), an additional 250‐mL bottle was filled with RO water in the laboratory prior to sampling and were transported alongside field sampling bottles to serve as a full‐process negative control.

### Sample processing and extraction

We placed water samples on ice for transport to the laboratory and then vacuum‐filtered, within 1 h, onto 1.2‐*μ*m pore‐sized polycarbonate membrane filters (EMD Millipore, Billerica, MA). We placed filters containing sample filtrates in 2.0‐mL microcentrifuge tubes containing 700 *μ*L of CTAB and stored at −20°C until extraction. DNA was extracted following a modified chloroform–isoamyl alcohol (24:1; Amresco, Dallas, TX) and an isopropanol precipitation of DNA (Renshaw et al. 2015): (1) the 2‐mL microcentrifuge tubes (filters and preservation buffer) were incubated in a 65°C water bath for 10 min; (2) 700 *μ*L of CI was added to each tube, and samples were vortexed for 5 sec; (3) tubes were centrifuged at room temperature at 18,400 g for 5 min, and 500 *μ*L of the aqueous layer was transferred to a fresh set of 2‐mL tubes; (4) 500 *μ*L of ice‐cold isopropanol and 250 *μ*L of 5mol/L NaCl were added to each tube, and samples were precipitated at −20°C overnight; (5) the precipitate was pelleted by centrifugation at room temperature at 18,400 g for 10 min, and the liquid was decanted; (6) 150 *μ*L of room temperature 70% ethanol was added to each tube to wash pellets; (7) tubes were centrifuged at 18,400 g for 5 min, and the liquid was decanted; (8) 150 *μ*L of room temperature 70% ethanol was added to each tube to wash pellets a second time; (9) tubes were centrifuged at 18,400 g for 5 min, and the liquid was decanted (10) pellets were dried in a vacufuge at 45°C for 15 min, followed by air‐drying until no visible liquid remained; and finally, (11) pellets were rehydrated with 100 *μ*L of 1X TE Buffer, low EDTA, and pH 8.0 (USB Corporation, Cleveland, OH). We treated resuspended DNA with the *OneStep™* PCR Inhibitor Removal Kit (Zymo Research, Irvine, CA) to remove potential inhibitors.

To observe potential artifacts such as contamination and errors from PCR, sequencing and bioinformatics, a single mock community sample was constructed (Schloss et al. 2011) and run through the DNA extraction process alongside eDNA samples. The mock community sample was composed of 60 ng of tissue‐derived DNA (measured with Qubit) from six Indo‐Pacific marine fishes: *Amphiprion ocellaris*,* Salarias fasciatus*,* Ecsenius bicolor*,* Centropyge bispinosa*,* Pseudanthias dispar*, and *Macropharyngodon negrosensis*. All four full‐process negative controls were extracted in a single batch separate from the eight eDNA samples, but followed the same protocols outlined above.

### PCR‐based Illumina library preparation and sequencing

We amplified four partial gene mitochondrial fragments: the Cytochrome B gene, two sections of the 12s gene, and 16s rDNA described in Evans et al. (2015). The first stage PCR was a 50‐*μ*L PCR for each of the four locus‐specific amplicons, a single reaction per sample per primer set. We used the following recipe: 29.5‐*μ*L sterile water, 10‐*μ*L 5× HF buffer, 1‐*μ*L 10‐mmol/L dNTPs, 1.5‐*μ*L 50‐mmol/L MgCl_2_, 1.25‐*μ*L 10‐*μ*mol/L forward primer, 1.25‐*μ*L 10‐*μ*mol/L reverse primer, 0.5‐*μ*L 2 U/*μ*L iProof High‐Fidelity DNA Polymerase (Bio‐Rad, Hercules, CA), and 5‐*μ*L DNA. Temperature cycling conditions were the same as outlined in Evans et al. (2015) for the L14735/H15149c, Ac12S, Am12S, and Ac16S primer sets.

To complete the Illumina sequencing library and individually barcode each sample, a 50‐*μ*L PCR was used for a second stage PCR, consisting of 22‐*μ*L sterile water, 10‐*μ*L 5× HF buffer, 1‐*μ*L 10‐mmol/L dNTPs, 1.5‐*μ*L 50‐mmol/L MgCl_2_, 5‐*μ*L 10‐*μ*mol/L Nextera Index Primer 1 (N701‐N712), 1.25‐*μ*L 10‐*μ*mol/L Nextera Index Primer 2 (S502‐S508 and S517), 0.5‐*μ*L 2 U/*μ*L iProof High‐Fidelity DNA Polymerase (Bio‐Rad), and 5‐*μ*L DNA. For the second stage PCR, the template DNA was a pool of 25 ng of DNA derived from all four markers from each sample in the following amounts: L14735/H15149c at 8 ng, Ac12s at 7.75 ng, Am12s at 3.25 ng, and Ac16s at 6 ng, and the total DNA volume brought to 5 *μ*L with the addition of sterile water. Adjusting for amount of total DNA allows larger fragments to amplify to the same extent as smaller fragments within the same library. Nextera Index Primers were synthesized by Integrated DNA Technologies (Coralville, IA) based on sequences available from Illumina, Inc. (San Diego, CA).

Temperature cycling conditions for the second stage PCR consisted of an initial denaturation step at 98°C for 2 min, followed by eight cycles of denaturation at 98°C for 10 sec, annealing at 55°C for 20 sec, and extension at 72°C for 30 sec, followed by a final extension step at 72°C for 10 min. The PCR Clean‐Up 2 protocol was followed (16S Metagenomic Sequencing Library Preparation), and DNA concentrations were quantified with the Qubit dsDNA HS Assay (Life Technologies, Carlsbad, CA). All four amplicon sizes were verified within each library on a Bioanalyzer DNA 7500 chip (Agilent Technologies, Santa Clara, CA). In addition to the eight eDNA samples from Juday Creek, we included the four full‐process negative controls, the single mock community, and four PCR no‐template controls (“NTCs”, sterile water in place of DNA template, one for each marker) in initial PCR stages.

We ran PCR products through a 2% agarose gel, stained with ethidium bromide, and visualized on a UV light platform. Amplified products were manually excised from the gels with single‐use razor blades, cleaned with the QIAquick Gel Extraction Kit (Qiagen, Venlo, the Netherlands), and eluted from spin columns with 30 *μ*L of Buffer EB. Although all NTCs failed to amplify per visual confirmation, we still excised a band from the agarose gel at the expected size for each NTC and carried through the remaining library preparation for subsequent Illumina sequencing per the recommendation of Nguyen et al. (2015). Two of the four full‐process controls showed amplification on the gel and were pooled with all other libraries for sequencing. We quantified the DNA concentration of each elution with the Qubit dsDNA HS Assay (Life Technologies). Libraries were pooled in equal molar concentrations along with PhiX (v3; Illumina) and paired‐end sequenced on an Illumina MiSeq in a single MiSeq flow cell by the University of Notre Dame's Genomics and Bioinformatics Core Facility (http://genomics.nd.edu/) with a MiSeq Reagent Kit v 3 (600‐cycle; Illumina).

### Bioinformatics analysis

**Figure 2:**
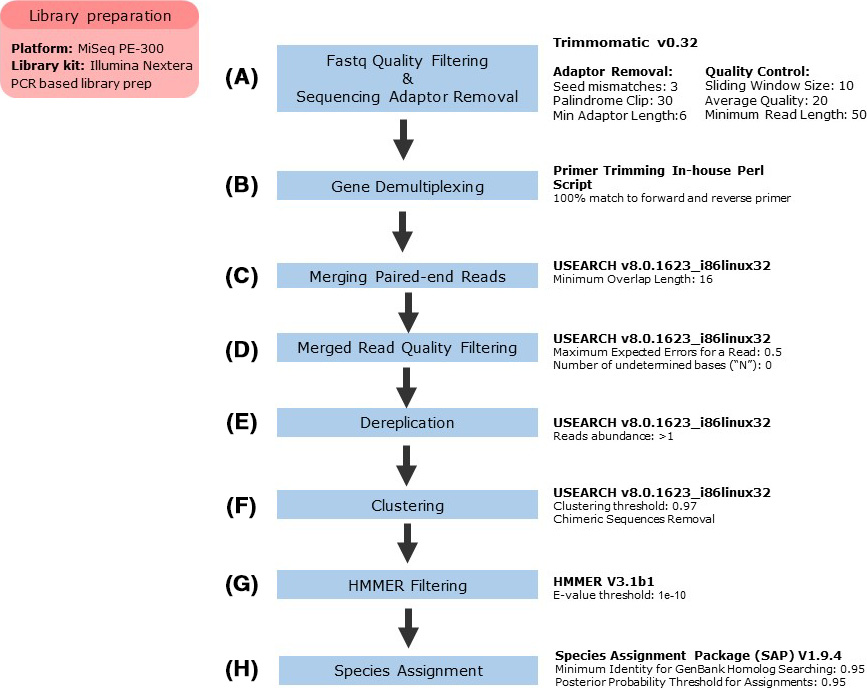
Flowchart describing bioinformatics steps taken to analyze MiSeq data. (A) Quality filtering is the process of removing low‐quality reads from further analysis. (B) Demultiplexing is the process of separating out individual samples that were run together on the same MiSeq run. (C) Merging is the process of combining overlapping reads into a single read. (D) Additional quality filtering step. (E) Dereplication is the process of eliminating duplicate reads. (F) Clustering is the process of grouping similar unique reads into OTUs. (G) HMMer is described in the manuscript. (H) SAP is described in the manuscript.

Raw sequence reads were filtered using Trimmomatic v0.32 to remove sequencing adaptor and low‐quality sequences with “ILLUMINACLIP: MiSeq.adapter.fas:3:30:6:1:true SLIDINGWINDOW:10:20: MINLEN:50” (Bolger et al. 2014). We removed reads with length <50 bp after trimming (see Fig. 2 for overview diagram of following methods). Paired‐end reads from the Illumina MiSeq were then split into four separate files based upon the forward and reverse primers unique to each specific mitochondrial amplicon, while retaining the integrity of each.fatsq file from read one and read two. We removed sequence reads without an exact match to their expected primer sequences and trimmed primer sequences from all reads. Overlapping paired‐end reads were then merged using USEARCH v8.0.1623 with default settings (Edgar 2010). We discarded reads with expected errors >0.5 or ambiguous base pairs at any nucleotide site. Finally, we pooled together the merged reads for all samples for each marker individually for operational taxonomic unit (OTU) generation.

### OTU generation and analysis

Using the program USEARCH v8.0.1623 (Edgar 2010), we removed singletons and dereplicated reads (Edgar 2013). We clustered OTUs based on a minimum sequence similarity of 97%, sorted from the largest cluster to the smallest. The sequence variant with the highest abundance in an OTU (based on pre‐dereplication counts) was chosen as the representative read for each OTU. We calculated the total read abundance for each OTU as the sum of all reads assigned to that cluster (i.e., all sequences with ≥97% nucleotide similarity to the representative read).

### HMMER filtering

To detect and eliminate nontarget OTUs, usually of bacterial origin, we applied a novel filtering step to distinguish nontarget OTUs with profile hidden Markov models using HMMER (Wheeler and Eddy 2013). To develop these HMMER models, we downloaded complete metazoan mitochondrion genomes from NCBI RefSeq Release 69. We used ecoPCR (Ficetola et al. 2010) to in silico cut mitochondrial genomes into target marker regions based on our four PCR primer sets. We allowed three mismatches in the primer regions and retained amplicon sizes within the 50‐ to 500‐bp range for further analysis. We then used OBITools to remove amplicon sequences that were identical between species (Boyer et al. 2015). We then aligned all amplicons that passed this filtering with Clustal Omega v1.2.0 (Goujon et al. 2010; Sievers et al. 2011) and built HMMER models based on the four alignments. To screen for nonmetazoan OTUs, we tested all our OTUs against the corresponding HMMER model. We treated OTUs with an *e*‐value <1e‐10 as target amplicons and retained them for species assignment.

### Species assignment

**Figure 3:**
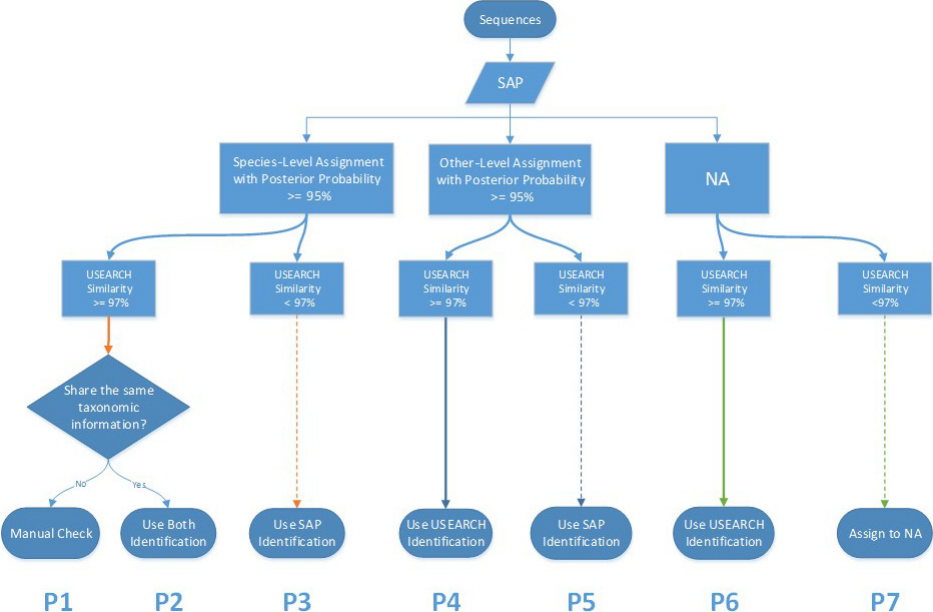
Decision‐making flowchart of species assignment utilizing OTU sequences inputted into SAP and USEARCH. For example, in the case of path 1 (P1): if SAP provides a species‐level assignment with posterior probability >=95%, USEARCH has a global alignment with identity >=97%, and the species assignments from the two approaches are identical, we use the species assignment. If the two assignments from each program are different (P2), we manually check the assignment against GenBank reference and alignment and make the decision as to the appropriate species assignment.

We used two different programs for species assignment, SAP v1.9.3 (Statistical Assignment Package; Munch et al. 2008a, 2008b) and USEARCH v8.0.1623 (Edgar 2010). First, we used SAP to assign OTUs with no a priori knowledge of existing species presence, utilizing all sequences found on the NR database of GenBank. SAP relies on the phylogeny of homologs found in the GenBank database; therefore, species with hybrids in the GenBank database are always assigned to a higher taxonomic level with low posterior probability, such as common carp (*Cyprinus carpio*). Therefore, we combined SAP results with a second method using a global alignment (USEARCH) based on a known reference list of species found in Juday Creek along with many related species for which we had tissues (Data available from the Dryad Digital Repository: http://dx.dooi.org/10.5061/dryad.d63sc: Table S1). We then made a consensus species assignment by comparing the assignment from SAP with the global identity by USEARCH to determine whether they matched. If not, we conducted a manual verification of the target sequence by comparing it to a reference sequence (see Fig. 3). OTUs that could not be assigned to species level were excluded from further analysis. We considered a species as present in a sample if we detected two or more reads in at least two or more markers.

### Species richness estimation

Observed species richness (*S*_obs_) is the number of species observed in a defined area surveyed as part of a study. However, what is directly observed does not likely capture the actual species richness of the area due to the effect of having sampled only a subset of the community (Colwell 2013). One approach to accounting for the unobserved species in an area is to apply an estimator, such as the Chao II estimator (Chao 2005), to find a lower bound, or minimum, of the species richness. These estimators are generally classified as abundance based or incidence based (Gotelli and Colwell 2011). Abundance‐based estimator use the rarity of individuals captured during a sampling effort. This would work for direct capture methods such as electrofishing, but not for eDNA approaches as we do not know the direct relationship between number of DNA sequences and number of individuals of a fish species. The alternative to abundance estimation is incidence‐based estimation where the frequency of detecting a species in an effort is used to estimate specie richness. In electrofishing, this is akin to having a species (potentially many individuals) detected in only one reach. With eDNA, this is akin to having eDNA from only one species in only one sample. To estimate the total number of unique species detected by electrofishing or eDNA, we applied the Chao II bias‐corrected estimator (Chao 2005; Colwell 2013). The Chao II estimates the minimum species richness in the system and accounts for unobserved species based on the sampling effort (*n*), number of species with only one incidence of detection, *g*(1), and the number of species with two incidences of detection *g*(2):

$$\hat{S}=S_{\mathrm{obs}}+\frac{n-1}{n}\times \frac{g(1)(g(1)-1)}{2(g(2)+1)}$$

where *S*_obs_ is the total number of species directly observed. The formulation of (*n *− 1)/*n* is a factor used to adjust for small sample size, which is needed for our samples of four and eight, in electrofishing and eDNA sampling, respectively. For example, if we had *n* = 10 electrofishing reaches, and we detected a total of *S*_obs_ = 20 unique species, and four species (*g*(1) = 4) were detected only in one reach, and three species were detected in only two reaches (*g*(2) = 3), then the expected species richness according to the bias‐corrected Chao II estimator would be

$$\hat{S}=20+\frac{10-1}{10}\times \frac{4(4-1)}{2(3+1)}=20+1.35=21.35.$$

The estimator indicates that there were 20 directly observed species in the overall effort, but that likely 1.35 species were not detected based on the incidence of rare species (number of species detected only in one reach (*g*(1)) or two reaches (*g*(2))) in the effort. We used a nonoverlap of unconditional 95% confidence intervals (Gotelli and Colwell 2011) as a conservative inference of significant difference (Colwell 2013). We calculated all species richness estimates and confidence intervals using EstimateS v9 (Colwell 2013).

### Risk of contamination

**Figure 4:**
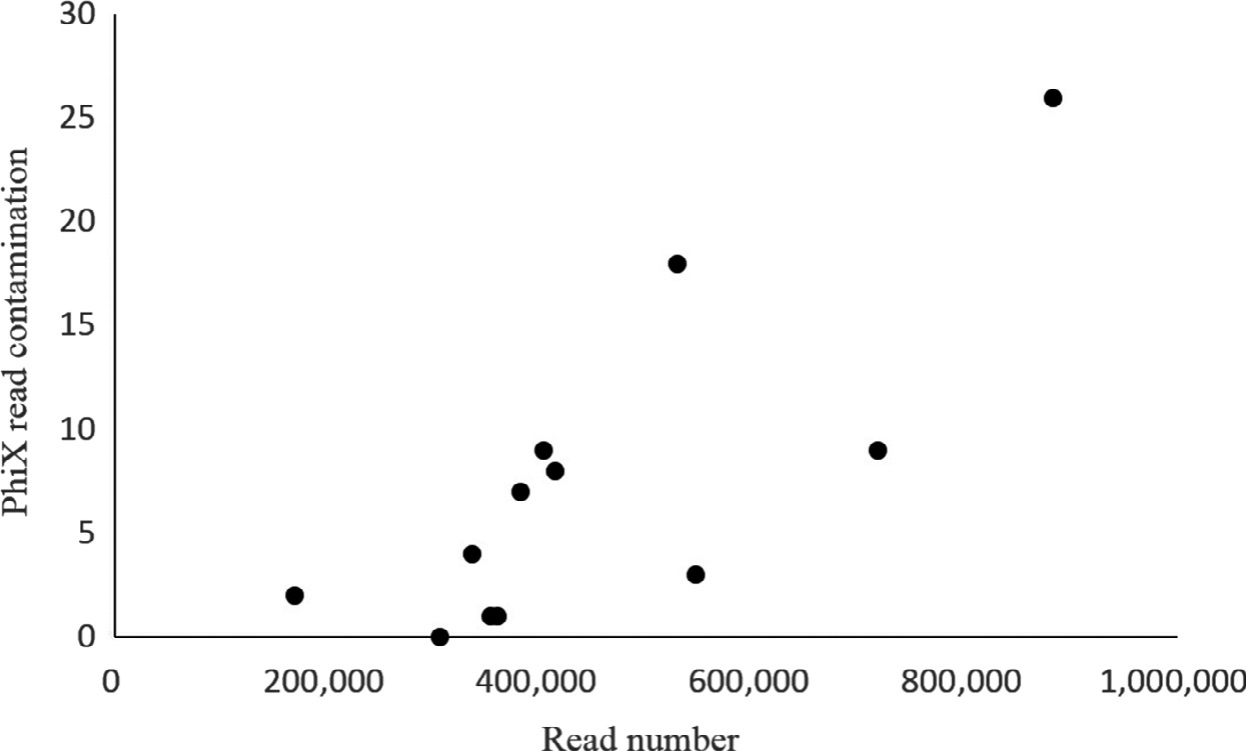
The number of PhiX control sequences that were incorrectly assigned to a sample as a function of the number of reads assigned to the sample. PhiX is an Illumina control library that is spiked into the pooled libraries prior to loading on the MiSeq. There are no laboratory steps or possibilities for contamination during preparation; therefore, any misassignment of this control is due to incorrect demultiplexing or index assignment due to cross‐contamination between clusters on the flow cell. This is therefore an instrument artifact and not likely due to any laboratory contamination, but demonstrates the plausibility that a low number of reads can be misassigned to any sample.

We assessed the contamination and its potential influence on our estimated species richness by fitting a Poisson distribution of contaminant DNA found in concurrently run negative controls, and then evaluated the probability that a sequence matching a particular species in each sample could have arisen by chance. We then flagged any species–marker combination with a probability >0.001 as a false positive detection due to contamination (Appendix S1: Table S2 in Supporting information). In cases where a sample had potential contamination for a marker, we re‐evaluated our conclusion about positive detection to reflect our rule of having at least two markers with at least two reads present and then recalculated species richness estimates for the rarefaction curve for comparison. Furthermore, we assessed potential cross‐library contamination by comparing the level of PhiX generated reads demultiplexed and assigned to each library (see Fig. 4 for full methods).

## Results

### Traditional sampling

**Figure 5:**
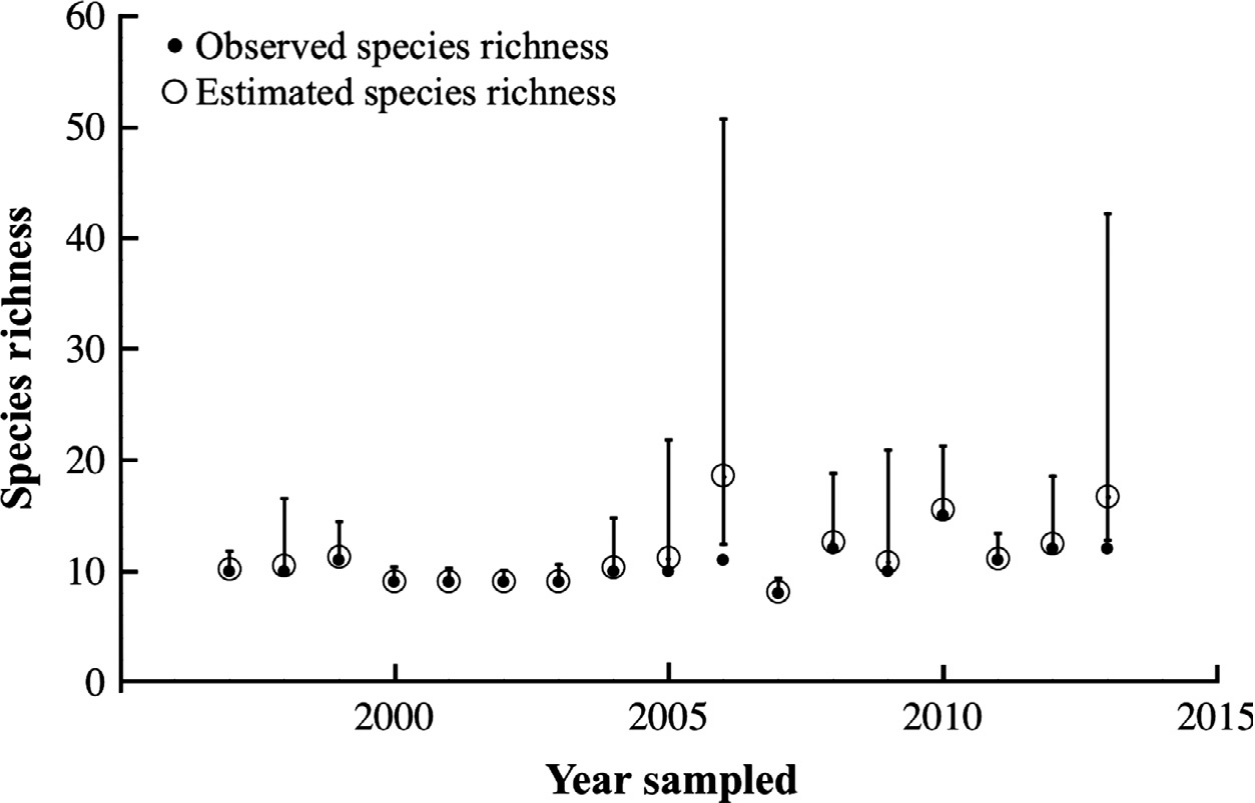
Species richness for Juday Creek from 1997 to 2013 for electrofishing for all four reaches combined, including species captured (black dots) and bias‐corrected Chao II species richness estimates (open circles) with 95% confidence intervals (vertical bars).

**Figure 6:**
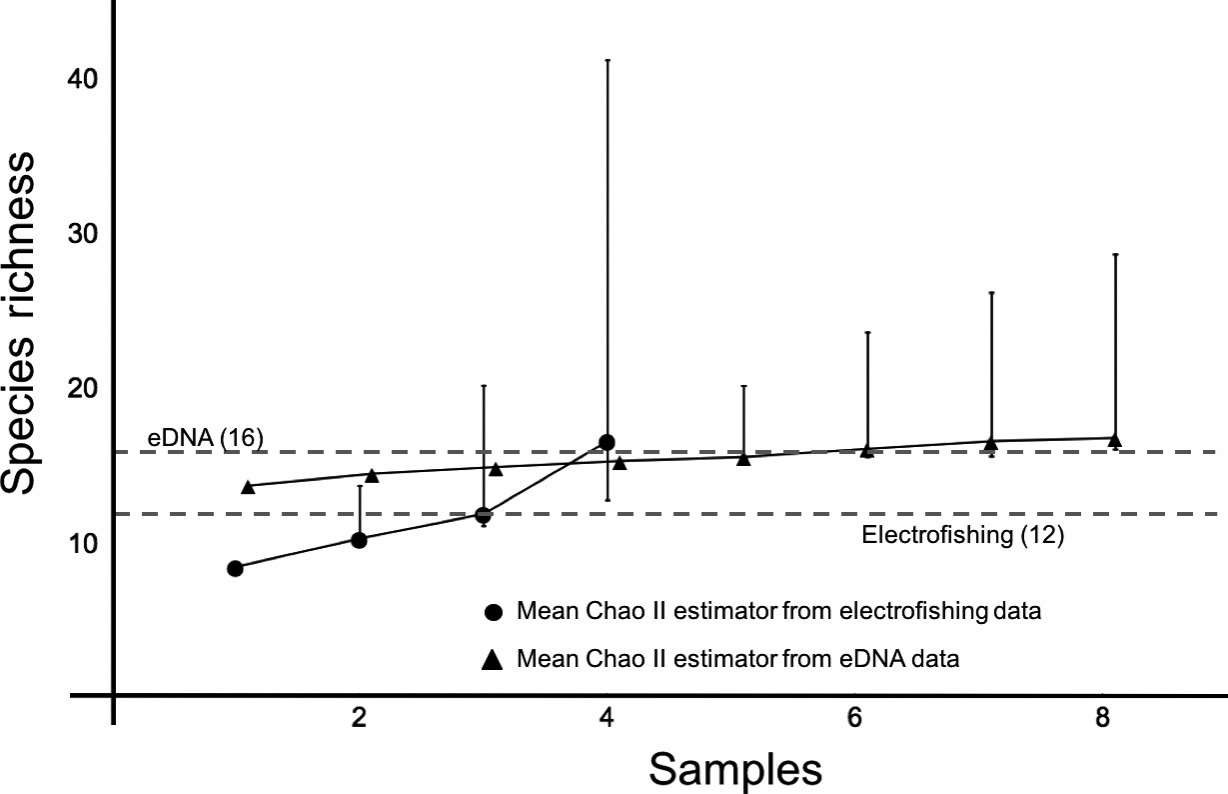
Species richness rarefaction curves (incidence based) for Juday Creek in 2013 for electroshocking (circles) and eDNA metabarcoding (triangles) samples. Vertical bars are 95% confidence intervals. Dashed lines represent observed values for the two methods. Absence of confidence intervals indicates that the estimated species richness is the same as the observed species richness.

In 2013, the 17th consecutive year of Juday Creek surveys, we caught a total of 12 fish species (Data available from the Dryad Digital Repository: http://dx.dooi.org/10.5061/dryad.d63sc: Table S3). Over the entire 17‐year span, 18 fish species in total were observed in the studied section of the stream during the annual sampling events (Table 1). Many species were found each year, including seasonally transient potadromous species (e.g., salmonids), while other species were rarely collected. The Chao II bias‐corrected estimators for the electrofishing effort applied over the 17‐year study indicates high consistency between detected and estimated species richness (Fig. 5). Notable exceptions were found in 2006 and 2013, when singleton captures (*g1;* only one fish captured) were frequent and resulted in a greater species richness estimate and wide confidence intervals around the Chao II bias‐corrected estimates. The 2013 electrofishing estimated species richness was 16.6 species present (cf. 12 species directly captured) with a confidence interval from 12.8 to 42.2 species (Fig. 6).

### eDNA metabarcoding

The eDNA metabarcoding approach detected 16 species in 2013, including all 12 directly observed by electrofishing plus four others: yellow bullhead (*Ameiurus natalis*), common carp (*C. carpio*), eastern mudminnow (*Umbra pygmaea*), and largemouth bass (*Micropterus salmoides*) (Table 2). The mean Chao II bias‐corrected estimate for the metabarcoding approach was equal to the 16 observed species, with the 95% confidence interval spanning 16.1–28.7 species (Fig. 6).

**Table 2:** Species detection (x) for electrofishing and metabarcoding methods by stream reach ordered from the most downstream reach (R1) to the most upstream reach (R4). Species detection by metabarcoding is defined as positive detection by at least two of the four markers (L14735/H15149c, Ac12s, Am12s, and Ac16s) in any single sample. D and U represent the downstream and upstream ends of each reach. Scientific names given in Table 1, with the exception of yellow bullhead (*Ameiurus natalis*), common carp (*Cyprinus carpio*), and eastern mudminnow (*Umbra pygmaea*)

Common name	Electrofishing method				Metabarcoding method using eDNA							
	R1	R2	R3	R4	R1 – D	R1 – U	R2 – D	R2 – U	R3 – D	R3 – U	R4 – D	R4 – U
Rock bass		x	x	x	x	x	x	x	x	x	x	x
Yellow bullhead					x	x	x	x	x	x	x	x
White sucker	x	x	x	x	x	x	x	x	x	x	x	x
Mottled sculpin	x	x	x	x	x	x	x	x	x	x	x	x
Common carp					x	x	x	x	x	x	x	x
Rainbow darter				x		x						
Johnny darter	x	x	x	x	x	x	x	x	x	x	x	x
Green sunfish	x	x	x	x	x	x	x	x	x	x	x	x
Bluegill sunfish	x					x	x	x	x	x	x	x
Smallmouth bass	x	x	x	x	x	x	x	x	x	x	x	x
Largemouth bass					x	x	x	x	x	x	x	x
Rainbow trout		x			x	x	x	x	x	x	x	x
Western blacknose dace	x		x	x	x		x	x	x	x	x	x
Brown trout	x				x		x	x				
Creek chub	x	x	x	x	x	x	x	x	x	x	x	x
Eastern mudminnow					x							

### High‐throughput sequencing statistics

We generated 5.4 million total reads from one Illumina MiSeq run. After primer demultiplexing, we retained 3 million reads (Data available from the Dryad Digital Repository: http://dx.dooi.org/10.5061/dryad.d63sc: Table S4). The demultiplexing rate was 74% for the Juday Creek samples and 27% for the control samples due to large amount of nonspecific amplicons in PCR negative controls and field blanks. A total of 47.1% of the raw reads passed the stringent filtering criteria. From the USEARCH analysis for OTUs on the combined pools of amplicon specific sequences, we detected 44, 30, 44, and 18 OTUs from Ac12s, Ac16s, Am12s, and L14735/H15149c markers, respectively (Data available from the Dryad Digital Repository: http://dx.dooi.org/10.5061/dryad.d63sc: Table S4).

### Species assignment

Based on SAP and USEARCH, we matched 24 OTUs with species‐level assignments to the marker Ac12s (including four mock community and six nonfish vertebrate species), 19 OTUs with species‐level assignments to the marker Ac16s (including three mock community), 22 OTUs with species‐level assignments to the marker Am12s (including six mock community and one nonfish vertebrate species), and 15 OTUs with species‐level assignments to the marker L14735/H15149c (including four mock community and two nonfish vertebrate species) (Data available from the Dryad Digital Repository: http://dx.dooi.org/10.5061/dryad.d63sc: Table S5). One OTU was assigned to the central mudminnow, *Umbra limi*, a species often captured with electrofishing, but was dropped from further consideration as it failed to meet the final filtering criteria of multiple markers. Additionally, six nonfish vertebrate species failed to meet the final filtering criteria of multiple markers and were subsequently dropped from further consideration (Data available from the Dryad Digital Repository: http://dx.dooi.org/10.5061/dryad.d63sc: red rows in Table S6). For Juday Creek, a total of 16 fish and one nonfish vertebrate species detections met our stringent bioinformatics criteria (Data available from the Dryad Digital Repository: http://dx.dooi.org/10.5061/dryad.d63sc: Table S6).

### Putative false positive and false negative rates

We detected nontarget OTUs using the HMMER models in three of the four amplicons. Three of 44 Ac12s OTUs, five of 30 Ac16s OTUs, one of 44 Am12s OTUs, and zero of 18 L14735/H15149c OTUs were detected as nontarget reads by HMMER (Data available from the Dryad Digital Repository: http://dx.dooi.org/10.5061/dryad.d63sc: Table S4). We also found several low abundance OTUs (1% of the total number of reads) that match a reference sequence with 90–96% similarity. These OTUs could be cryptic species, rare haplotypes within the population, or false positive OTUs due to PCR errors. Four of 44 of the Ac12s OTUs, six of 30 Ac16s OTUs, 17 of 44 of Am12s OTUs, and one of 18 of L14735/H15149c OTUs fell into this category. We manually checked all OTUs that had a closely related OTU (90–96.9% similarity) against NCBI GenBank. None of these OTUs matched a more similar sequence on NCBI GenBank. A possible explanation is that these OTUs were not real haplotypes, but PCR errors from the correct templates.

### Positive and negative controls

From the sequencing of the mock community, we detected all six marine fish and five additional species of fish that were also present in the Juday Creek reach samples. In the PCR negative control, we detected all six of the mock community marine fish and nine additional fish species found in the Juday Creek reach samples. In field blanks 1 and 2, we also detected two and three mock community marine fishes and seven and nine additional fish species found in the Juday Creek reach samples, respectively. Human DNA was also found in the field blank samples (Data available from the Dryad Digital Repository: http://dx.dooi.org/10.5061/dryad.d63sc: Table S7).

We detected measurable DNA in our negative controls, and our assessment of possible contamination resulted in 20 instances needing further evaluation. Of these 20 instances, only three changes in detection occurred because of possible contamination (Appendix S1). One sample from bluegill and two samples from yellow bullhead changed from positive detection to no detection. However, our re‐evaluation of species richness and the species accumulation curve is unchanged, maintaining our initial conclusion of 16 species detected using eDNA metabarcoding (Appendix S1).

## Discussion

### Ecological implications of sampling approach

Knowledge of natural ecosystem biodiversity is fundamental to assessing ecosystem function and the ongoing or predicted impacts of environmental change (Butchart et al. 2010). Freshwater environments represent a small fraction of the Earth's area, yet harbor a disproportionately large amount of global biodiversity (Strayer and Dudgeon 2010) undergoing measurable losses (Vörösmarty et al. 2010). Estimation of species richness in any ecological setting and especially in aquatic environments can be challenging due to the rareness of some species (Gu and Swihart 2004), variable detection probabilities (MacKenzie et al. 2002), and the field effort necessary to collect sufficient samples or species to ensure meaningful coverage (Gotelli and Colwell 2011). With a traditional sampling method (e.g., electrofishing), we maximized our probability of detecting fish species by sampling a small stream intensively at multiple sites over multiple years. eDNA sampling detected all species captured by electrofishing, and also detected additional species putatively predicted to be present based on Chao II estimation from electrofishing data. At the same time, eDNA sampling substantially reduced stress on captured fish and disruption of stream habitat. The consistency of observed species richness estimates from eDNA and electrofishing, coupled with the added benefits of identifying additional species without increased effort and harassment, represents a transformative method for biomonitoring of fish fauna (Evans et al. 2015; Adamson and Hurwood 2016).

Detection of uncommon, rare, or elusive species is critically important for accurate estimates of species richness and is often a priority for resource managers (McDonald 2004), particularly if those species are incipient invaders or species of conservation concern (Lodge et al. 2012). The difference in observed species richness between electrofishing (12) and metabarcoding applied to eDNA samples (16) is intriguing, but predicted by the Chao II estimator from electrofishing data. It is possible that we needed to conduct more electrofishing to capture the additional species, but we applied intensive triple‐pass electrofishing effort to the system. Even with further electrofishing effort, we would only expect to capture previously recorded fish, such as the (uncommon) largemouth bass. In this instance, three species with benthic life‐history strategies (common carp, eastern mudminnow, and yellow bullhead) that had never been captured during the annual electrofishing surveys were detected by eDNA. The result suggests that eDNA metabarcoding may improve detection probabilities for benthic species that can be difficult to efficiently capture with electrofishing (Fisher 1987; Reyjol et al. 2005).

A strong possibility is that some of the eDNA we sampled emanated from species that occur in reaches upstream from those we sampled. The four reaches sampled using electrofishing were an aggregate 240 m in length, spanning about 1,200 m of stream, but DNA can persist in the environment for days (Dejean et al. 2011) and can be transported meters to kilometers away from its source (Deiner and Altermatt 2014). One reasonable explanation for detection of species not directly captured is this downstream transport of eDNA into an area where the species are locally absent. Every environment and set of conditions will have different DNA degradation rates (Barnes et al. 2014) and changing flow dynamics in the case of streams. The detection of carp eDNA in our samples is likely due to the presence of carp or koi (i.e., domesticated common carp) in backyard ponds physically connected to Juday Creek at upstream locations. Additionally, carp are known to inhabit the St. Joseph River into which Juday Creek drains (Deegan 2011). While suitable carp habitat is not in our sampling areas, suitable habitat does exist upstream, including large in‐channel ponds that could support common carp and other species preferring slow‐moving water, as well as that for all the noncaptured species detected by eDNA. Thus, our study is insufficient to distinguish between the possibility that some species were present in the stream reaches, but not captured by electrofishing and the possibility that the species detected occurred only in parts of the watershed upstream from our sampling reaches.

Added sensitivity to detect unobserved, but potentially upstream or locally absent species becomes advantageous as the geographic scale of eDNA metabarcoding increases. Because species can occur over vast geographic ranges and habitat types, scalability of detection becomes an issue for traditional methods. Moving from small streams to large rivers or lakes requires different techniques and expensive equipment. eDNA sampling methods can be utilized in all types of habitats with the same equipment, allowing for surveys across wide geographic ranges with minimal increase in effort or cost. Indeed, the ability to characterize the species richness by watershed using eDNA may be the most appropriate scale for future inferences, but this ability to characterize species richness must be determined by the transport dynamics (Deiner and Altermatt 2014; Deiner et al. 2015) and ecology of eDNA (Barnes et al. 2014).

The metabarcoding eDNA approach also differs from direct capture in that it uses genetics as opposed to morphology to identify species, which may be valuable when morphologically similar species are present in a system (e.g., central mudminnow vs. eastern mudminnow). We detected eastern mudminnow in our eDNA samples, but to our knowledge, only the central mudminnow is found in Juday Creek and Indiana in general. This mismatch may be due to inaccurate genetic data stored in GenBank (e.g., central mudminnow sequences logged as eastern mudminnow), inaccurate range data for mudminnows, hybridization between cryptic species, errors in alpha taxonomy, or misidentifications in the field. We recommend exercising caution when using a single method to infer presence of a species in combination with leveraging any historical or local expert knowledge to strengthen the argument for the presence of a novel species. For example, largemouth bass and yellow bullhead were previously captured in Juday Creek using electrofishing, and yellow bullhead and common carp are found in the region and likely occur in the Juday Creek watershed (Deegan 2011).

### Limitations of eDNA metabarcoding

Environmental DNA metabarcoding can introduce opportunities for false positive detection through molecular procedures within the laboratory and bioinformatics analysis. To mitigate these errors, stringent criteria are needed to increase confidence in a positive detection from a small fragment of DNA. In addition to separating pre‐ and post‐PCR laboratory procedures, we employed a requirement of multiple reads across multiple markers within each sample for a positive detection. While this criterion increases our confidence that a single incidence of contamination within a sample or errors due to PCR amplification would not be found across multiple extraction, PCR, and sequencing steps, recent data suggest that contamination can occur between libraries within the same sequencing run of high‐throughput machines such as the Illumina MiSeq (Mukherjee et al. 2015).

Using signatures of PhiX, a bacteriophage genome that is used as a control sequence added to the pooled libraries just prior to injection on the MiSeq, we detected low levels of contamination in our data (0.01–0.03% PhiX; Fig. 4). These rates are similar to those found in false index pairs with sequence reads due to mixed clusters on the flow cell (Kircher et al. 2012). Our data suggest that with this low level of cross‐contamination, our overall interpretation does not change due to the power of assessing the community with multiple genetic markers (Appendix S1). The cross‐library contamination found when multiple libraries are run on the same flow cell warrants further investigation (Mukherjee et al. 2015). Researchers should be aware, however, that a low number of reads assigned to a library during the demultiplexing of libraries could be artifacts. We found a correlation in our data that suggests when another library run on the same flow cell has a high number of reads for that species this tends to cause a low level of contamination in all other libraries (Appendix S1). Until either upgrades to the Illumina MiSeq platform or bioinformatic solutions are proposed, this low level of library‐to‐library contamination remains a significant challenge for eDNA metabarcoding studies.

Contamination is a critical issue in estimating species richness from eDNA using high‐throughput sequencing (Murray et al. 2015), as false detections will lead to smaller confidence intervals on species accumulation curves and has the potential to overestimate the number of undetected species present in the system. We identified three instances (bluegill in sample R1 and yellow bullhead in samples R5 and R7) where contamination likely led us to incorrectly conclude a detection occurred. These three instances of plausible contamination occurred for species having detections in all other samples, and thus, there was no discernable change to our Chao estimate and confidence in species richness. In all three of our possible contaminations, one marker had no indication of contamination. Because we required each species sample combination to have at least two observed sequences in two markers, we ultimately considered these samples to be nondetections. Using four markers to determine presence improved our assessment of species presence and reliability of our conclusions about species richness. Further, refining the number of markers used and the error distribution of contaminant sequences is a necessary avenue of research for providing more robust evaluation of species richness using eDNA.

## Conclusions

We demonstrate that eDNA‐based estimates of species richness of stream fish assemblages can provide a valuable improvement to capture‐based approaches. The eDNA approach also entails minimal harassment to fishes or impact on their habitat. Currently, however, eDNA cannot provide data on populations (e.g., abundance, biomass) or individuals (e.g., sex, weight, length, condition) quantifiable by direct capture. We also demonstrate with a species richness estimation model that the predicted, but unnamed, species from capture‐based approaches can be observed and named from their DNA in the environment. Our findings suggest that eDNA metabarcoding can improve the accuracy of species detection for aquatic environments and will be a transformative tool for monitoring aquatic biodiversity on a changing planet.

## Data Accessibility

- Raw reads from Illumina MiSeq run have been submitted to NCBI (BioSample ID: SRS1397719).
- Centroid sequences for each identified OTU and supplemental tables have been submitted to Dryad (doi:10.5061/dryad.d63sc)

## Conflict of Interest

None declared.

## Supporting information


**Appendix S1.** Estimating contamination rates and assessing false positives.
**Table S1**. Parameter estimates (*λ*) and associated probabilities that the observed number of Johnny Darter sequences in field samples came from the distribution of errant DNA.
**Table S2**. Detection of species as a result of presence of DNA in samples and consideration of contamination.
**Table S3.** Comparison of mean Chao estimator and confidence interval for incidence base accumulation curve without and with contamination considered.

**Table S4.** Number of reads from each sample (i.e. library) run on the Illumina MiSeq platform for each step of the bioinformatic pipeline. Samples are ordered from the most downstream sample (R1) to the most upstream sample (R8).

**Table S5.** Results for all species identified with OTU species assignment. Values of P1 – P7 represent the pathway taken to arrive at species assignment using SAP and USearch (Fig. S1).

**Table S6.** Raw read counts of each species identified, for each sample and all four markers. Samples are ordered from the most downstream sample (R1) to the most upstream sample (R8). Rows idenitfied in red were dropped from further analysis.

**Table S7.** Number of raw reads assigned to each species in control samples. Species considered present after all bioinformatic filtering and species assignment thresholds applied in negative controls. The eDNA column shows the species detected in the actual Juday Creek samples from Table S6. The P/A columns shows presence (1) or absence (0) given our criteria of >1 read and reads in multiple markers or samples.

## References

[ece32186-bib-0001] Adamson, E. A. , and D. A. Hurwood . 2016 Molecular ecology and stock identification. pp. 811–829 in J. F. Craig , ed. freshwater fish ecologyl. John Wiley & Sons, Ltd, Chichester, UK.

[ece32186-bib-0002] Arponen, A. , R. K. Heikkinen , C. D. Thomas , and A. Moilanen . 2005 The value of biodiversity in reserve selection: representation, species weighting, and benefit functions. Conserv. Biol. 19:2009–2014.

[ece32186-bib-0003] Barnes, M. A. , C. R. Turner , C. L. Jerde , M. A. Renshaw , W. L. Chadderton , and D. M. Lodge . 2014 Environmental conditions influence eDNA persistence in aquatic systems. Environ. Sci. Technol. 48:1819–1827. 24422450 10.1021/es404734p

[ece32186-bib-0004] Bolger, A. M. , M. Lohse , and B. Usadel . 2014 Trimmomatic: a flexible trimmer for Illumina sequence data. Bioinformatics. 30:2114–2120. 24695404 10.1093/bioinformatics/btu170 PMC4103590

[ece32186-bib-0005] Boyer, F. , C. Mercier , A. Bonin , Y. Le Bras , P. Taberlet , and E. Coissac . 2015 obitools: a unix‐inspired software package for DNA metabarcoding. Mol. Ecol. Resour. 16:176–182. doi:10.1111/1755‐0998.12428. 25959493 10.1111/1755-0998.12428

[ece32186-bib-0006] Butchart, S. H. , M. Walpole , B. Collen , A. Van Strien , J. P. Scharlemann , R. E. Almond , et al. 2010 Global biodiversity: indicators of recent declines. Science 328:1164–1168. 20430971 10.1126/science.1187512

[ece32186-bib-0007] Chao, A. 2005 Species richness estimation Pp. 7909–7916 *in* Balakrishnan N., Read C. and Vidakovic B., eds. Encyclopedia of statistical sciences. Wiley, New York.

[ece32186-bib-0008] Chao, A. , R. K. Colwell , C.‐W. Lin , and N. J. Gotelli . 2009 Sufficient sampling for asymptotic minimum species richness estimators. Ecology 90:1125–1133. 19449706 10.1890/07-2147.1

[ece32186-bib-0009] Colwell, R. K. 2013 EstimateS: Statistical estimation of species richness and shared species from samples. Available at: http://purl.oclc.org/estimates. Accessed October 25, 2015.

[ece32186-bib-0010] Deegan, D. 2011 Elkhart – south bend aquatic community monitoring. Pp. 85 City of Elkhart Public Works and Utilities, South Bend, IN.

[ece32186-bib-0011] Deiner, K. , and F. Altermatt . 2014 Transport distance of invertebrate environmental DNA in a natural river. PLoS One 9:e88786. 24523940 10.1371/journal.pone.0088786 PMC3921251

[ece32186-bib-0012] Deiner, K. , E. A. Fronhofer , E. Mächler , and F. Altermatt . 2015 Environmental DNA reveals that rivers are conveyer belts of biodiversity information. bioRxiv 2015:020800. doi: 10.1101/020800. 10.1038/ncomms12544 PMC5013555 27572523

[ece32186-bib-0013] Dejean, T. , A. Valentini , A. Duparc , S. Pellier‐Cuit , F. Pompanon , P. Taberlet , et al. 2011 Persistence of environmental DNA in freshwater ecosystems. PLoS One 6:e23398. 21858099 10.1371/journal.pone.0023398 PMC3152572

[ece32186-bib-0014] Didham, R. K. , J. M. Tylianakis , N. J. Gemmell , T. A. Rand , and R. M. Ewers . 2007 Interactive effects of habitat modification and species invasion on native species decline. Trends Ecol. Evol. 22:489–496. 17673330 10.1016/j.tree.2007.07.001

[ece32186-bib-0015] Edgar, R. C. 2010 Search and clustering orders of magnitude faster than BLAST. Bioinformatics 26:2460–2461. 20709691 10.1093/bioinformatics/btq461

[ece32186-bib-0016] Edgar, R. C. 2013 UPARSE: highly accurate OTU sequences from microbial amplicon reads. Nat. Methods 10:996–998. 23955772 10.1038/nmeth.2604

[ece32186-bib-0017] Evans, N. T. , B. P. Olds , C. R. Turner , M. A. Renshaw , Y. Li , C. L. Jerde , et al. 2015 Quantification of mesocosm fish and amphibian species diversity via eDNA metabarcoding. Mol. Ecol. Resour. 16:29–41. doi:10.1111/1755‐0998.12433. 26032773 10.1111/1755-0998.12433 PMC4744776

[ece32186-bib-0018] Ficetola, G. F. , E. Coissac , S. Zundel , T. Riaz , W. Shehzad , J. Bessière , et al. 2010 An in silico approach for the evaluation of DNA barcodes. BMC Genom. 11:434. 10.1186/1471-2164-11-434 PMC3091633 20637073

[ece32186-bib-0019] Fisher, W. L. 1987 Benthic fish sampler for use in riffle habitats. Trans. Am. Fish. Soc. 116:768–772.

[ece32186-bib-0020] Fukumoto, S. , A. Ushimaru , and T. Minamoto . 2015 A basin‐scale application of environmental DNA assessment for rare endemic species and closely related exotic species in rivers: a case study of giant salamanders in Japan. J. Appl. Ecol. 52:358–365.

[ece32186-bib-0021] Gotelli, N. J. , and R. K. Colwell . 2011 Estimating species richness Pp. 39–54 *in* Magurran A. E. and McGill B. J., eds. Frontiers in measuring biodiversity. Oxford Univ. Press, New York.

[ece32186-bib-0022] Goujon, M. , H. McWilliam , W. Li , F. Valentin , S. Squizzato , J. Paern , et al. 2010 A new bioinformatics analysis tools framework at EMBL–EBI. Nucleic Acids Res. 38:W695–W699. 20439314 10.1093/nar/gkq313 PMC2896090

[ece32186-bib-0023] Gu, W. , and R. K. Swihart . 2004 Absent or undetected? Effects of non‐detection of species occurrence on wildlife–habitat models. Biol. Conserv. 116:195–203.

[ece32186-bib-0024] Kircher, M. , S. Sawyer , and M. Meyer . 2012 Double indexing overcomes inaccuracies in multiplex sequencing on the Illumina platform. Nucleic Acids Res. 40:e3. 22021376 10.1093/nar/gkr771 PMC3245947

[ece32186-bib-0025] Lodge, D. M. , C. R. Turner , C. L. Jerde , M. A. Barnes , L. Chadderton , S. P. Egan , et al. 2012 Conservation in a cup of water: estimating biodiversity and population abundance from environmental DNA. Mol. Ecol. 21:2555–2558. 22624944 10.1111/j.1365-294X.2012.05600.x PMC3412215

[ece32186-bib-0026] Mace, G. M. , K. Norris , and A. H. Fitter . 2012 Biodiversity and ecosystem services: a multilayered relationship. Trends Ecol. Evol. 27:19–26. 21943703 10.1016/j.tree.2011.08.006

[ece32186-bib-0027] MacKenzie, D. I. , J. D. Nichols , G. B. Lachman , S. Droege , J. Andrew Royle , and C. A. Langtimm . 2002 Estimating site occupancy rates when detection probabilities are less than one. Ecology 83:2248–2255.

[ece32186-bib-0028] Mahon, A. R. , L. R. Nathan , and C. L. Jerde . 2014 Meta‐genomic surveillance of invasive species in the bait trade. Conserv. Genet. Resour. 6:563–567.

[ece32186-bib-0029] McDonald, L. L. 2004 Sampling rare populations Pp. 11–42 *in* Thompson W. L., ed. Sampling rare or elusive species: concepts, designs, and techniques for estimating population parameters. Island Press, Washington, DC.

[ece32186-bib-0030] Moerke, A. H. , and G. A. Lamberti . 2003 Responses in fish community structure to restoration of two Indiana streams. N. Am. J. Fish. Manag. 23:748–759.

[ece32186-bib-0031] Mukherjee, S. , M. Huntemann , N. Ivanova , N. C. Kyrpides , and A. Pati . 2015 Large‐scale contamination of microbial isolate genomes by Illumina PhiX control. Stand. Genomic Sci. 10:18. 26203331 10.1186/1944-3277-10-18 PMC4511556

[ece32186-bib-0032] Munch, K. , W. Boomsma , J. P. Huelsenbeck , E. Willerslev , and R. Nielsen . 2008a Statistical assignment of DNA sequences using Bayesian phylogenetics. Syst. Biol. 57:750–757. 18853361 10.1080/10635150802422316

[ece32186-bib-0033] Munch, K. , W. Boomsma , E. Willerslev , and R. Nielsen . 2008b Fast phylogenetic DNA barcoding. Philos. Trans. R. Soc. Lond. B Biol. Sci. 363:3997–4002. 18852104 10.1098/rstb.2008.0169 PMC2607413

[ece32186-bib-0034] Murray, D. C. , M. L. Coghlan , and M. Bunce . 2015 From benchtop to desktop: important considerations when designing amplicon sequencing workflows. PLoS One 10:e0124671. 25902146 10.1371/journal.pone.0124671 PMC4406758

[ece32186-bib-0035] Nguyen, N. H. , D. Smith , K. Peay , and P. Kennedy . 2015 Parsing ecological signal from noise in next generation amplicon sequencing. New Phytol. 205:1389–1393. 24985885 10.1111/nph.12923

[ece32186-bib-0036] Renshaw, M. A. , B. P. Olds , C. L. Jerde , M. M. McVeigh , and D. M. Lodge . 2015 The room temperature preservation of filtered environmental DNA samples and assimilation into a phenol–chloroform–isoamyl alcohol DNA extraction. Mol. Ecol. Resour. 15:168–176. 24834966 10.1111/1755-0998.12281 PMC4312482

[ece32186-bib-0037] Reyjol, Y. , G. Loot , and S. Lek . 2005 Estimating sampling bias when using electrofishing to catch stone loach. J. Fish Biol. 66:589–591.

[ece32186-bib-0038] Risser, P. G. 1995 Biodiversity and ecosystem function. Conserv. Biol. 9:742–746.

[ece32186-bib-0039] Schloss, P. D. , D. Gevers , and S. L. Westcott . 2011 Reducing the effects of PCR amplification and sequencing artifacts on 16S rRNA‐based studies. PLoS One 6:e27310. 22194782 10.1371/journal.pone.0027310 PMC3237409

[ece32186-bib-0040] Sievers, F. , A. Wilm , D. Dineen , T. J. Gibson , K. Karplus , W. Li , et al. 2011 Fast, scalable generation of high‐quality protein multiple sequence alignments using Clustal Omega. Mol. Syst. Biol. 7:539. 21988835 10.1038/msb.2011.75 PMC3261699

[ece32186-bib-0041] Simmons, M. , A. Tucker , W. L. Chadderton , C. L. Jerde , A. R. Mahon , and E. Taylor . 2015 Active and passive environmental DNA surveillance of aquatic invasive species. Can. J. Fish Aquat. Sci. 73:1–8.

[ece32186-bib-0042] Strayer, D. L. , and D. Dudgeon . 2010 Freshwater biodiversity conservation: recent progress and future challenges. J. N. Am. Benthol. Soc. 29:344–358.

[ece32186-bib-0043] Thomsen, P. F. , and E. Willerslev . 2015 Environmental DNA – an emerging tool in conservation for monitoring past and present biodiversity. Biol. Conserv. 183:4–18.

[ece32186-bib-0044] Thomsen, P. F. , J. Kielgast , L. L. Iversen , C. Wiuf , M. Rasmussen , M. T. P. Gilbert , et al. 2012 Monitoring endangered freshwater biodiversity using environmental DNA. Mol. Ecol. 21:2565–2573. 22151771 10.1111/j.1365-294X.2011.05418.x

[ece32186-bib-0045] Turner, W. , S. Spector , N. Gardiner , M. Fladeland , E. Sterling , and M. Steininger . 2003 Remote sensing for biodiversity science and conservation. Trends Ecol. Evol. 18:306–314.

[ece32186-bib-0046] Valentini, A. , P. Taberlet , C. Miaud , R. Civade , J. Herder , P. F. Thomsen , et al. 2015 Next‐generation monitoring of aquatic biodiversity using environmental DNA metabarcoding. Mol. Ecol. 25:929–942. doi:10.1111/mec.13428. 26479867 10.1111/mec.13428

[ece32186-bib-0047] Vörösmarty, C. J. , P. McIntyre , M. O. Gessner , D. Dudgeon , A. Prusevich , P. Green , et al. 2010 Global threats to human water security and river biodiversity. Nature 467:555–561. 20882010 10.1038/nature09440

[ece32186-bib-0048] Wheeler, T. J. , and S. R. Eddy . 2013 nhmmer: DNA homology search with profile HMMs. Bioinformatics. 29:2487–2489. 23842809 10.1093/bioinformatics/btt403 PMC3777106

