# Supplementary material for: Estimating species richness using environmental DNA
Source: Ecol Evol. 2016 May 30;6(12):4214–26. doi: 10.1002/ece3.2186 (PMC4972244; doi:10.1002/ece3.2186)
Supplement: Supplementary file 1 — Appendix S1. Estimating contamination rates and assessing false positives. Table S1. Parameter estimates (λ) and associated probabilities that the observed number of Johnny Darter sequences in field samples came from the distribution of errant DNA. Table S2. Detection of species as a result of presence of DNA in samples and consideration of contamination. Table S3. Comparison of mean Chao estimator and confidence interval for incidence base accumulation curve without and with contamination considered. [file ECE3-6-4214-s001.docx]

**Appendix 1: Estimating contamination rates and assessing false positives for detection of fishes with eDNA in Juday Creek, Indiana, USA.**

The use of quality controls are extremely important in high-throughput eDNA metabarcoding studies to investigate artifacts such as background contamination and recovery of expected DNA through bioinformatics workflows (Murray et al 2015). In our study of Juday Creek, five such quality controls were included during the laboratory processing and high-throughput sequencing (HTS) of eDNA samples. We included two full process, “cooler blanks” consisted of reverse osmosis water filled in a sampling container in the lab and transported during field collections (one on each of the two sampling days). These cooler blanks where subsequently filtered and extracted along-side samples from Juday Creek and used in PCR through to library preparation and HTS. The third control was a PCR negative control that was used at the first step of PCR and carried through library preparation and HTS. Fourth, a positive PCR control that consisted of a pool of DNA extracted from tissues of tropical marine fish not known to occur in the area and were PCR-amplified alongside field collected samples to create a mock community. These four controls were then used to monitor for contamination during the laboratory phase during the study. A fifth control, known as PhiX, was added as a control to the libraries before running on the MiSeq for HTS. This control, and its results, are additionally discussed in the Supplemental Fig. 2 and will not be considered further here.

A total of 16 species were identified from field samples using four different markers for a total of 64 marker-species combinations, each having four potential observations that could be observed in controls. If there was no contamination in any negative control, then we would expect to see no sequences from these 16 species in any of the four markers. However, in 46% of control observations (119 of 256), one or more sequences were assigned to a marker-species combination when there should have been zero sequences. On average, there were 18 sequences per marker-species combination across the four controls (*n*=256, Std. Dev. = 76). There was a significant difference among markers (GLM; Poisson error with log-link function, *p*<0.01). Actino12s marker averaged 45 sequences per marker-species combination (Std. Dev. = 141), while Actino 16s, Amphibia12s and Cyt B averaged 3 (Std. Dev. = 8), 8 (Std. Dev. = 23), and 14 (Std. Dev. = 42), respectively.

With increasing amounts of sequences observed for a given species in a field sample, it may be expected that there is increased potential for DNA to contaminate a control. To test this hypothesis, we evaluated correlations between the total number of sequences observed per species for each marker in field samples with the number of sequences per species for each marker in each of the four control samples. The correlation was significantly positive for all markers: Actino12s (*r*=0.98, *p<*0.01), Actino16s (*r*=0.93, *p<*0.01), Amphibia12S (*r*=0.99, *p<*0.01), and CytB (*r*=0.99, *p<*0.01). As such, there is evidence that the error distribution of the amount of contaminant DNA is unique to each marker-species combination and is correlated to the observed number of sequences for that species and marker in the field controls.

Since the data are represented in counts of DNA sequences per sample, we can assume each of the four control observations are from a Poisson distribution that describes the number of errant sequences that we should expect to see in any given quality control. The maximum likelihood estimator for the mean of the Poisson distribution is $\hat{\lambda}=\frac{\sum x_{i}}{n}$. While other count data distributions may be better suited, such as negative binomial (McMurdie & Holmes 2014), there is little empirical evidence to support using these distributions at this time and with only four data points, estimating more than one parameter for the error distribution is difficult to justify. We choose to move forward working on the assumption of a Poisson error distribution and recognize the need for more research to better justify this assumption – presumably from a study with more quality controls simultaneously collected and analyzed. Below we walk through this logic applied to an example with the fish Johnny Darter and enumerate this for all other species in Supplemental Table S2 to test whether low levels of contamination, no matter their source, can influence the outcome of biological interpretation for the species we detect in Juday Creek. We used a stringent threshold of more than two sequences observed for at least two markers to consider a species present at a field site. In addition, we chose to use a statistical approach rather than the arbitrary removal of sequences from the whole dataset as currently practiced (e.g., Valentini et al. 2015), to ask the question at this threshold what is the likelihood of concluding a species is at a site when it in in fact not.

For example, the Johnny Darter (*Etheostoma nigrum*) had sequences numbers of 11,4, 81, and 168 (Table S7) for the Ac12s marker observed in the four quality controls. These counts are the number of sequences from samples that should have no sequences present and therefore represent background levels of contamination during our eDNA assay. The mean number of copies as estimated from the Poisson distribution is $\hat{\lambda}=66$. There were eight samples collected and processed from Juday Creek and each sample had a number of sequences observed for the Ac12s marker for Johnny Darter, {355, 457, 1062, 512, 694, 1587, 2264, 2089} (Table S6). Given our estimated distribution of errant Ac12s Johnny Darter sequences in the quality controls, we can use the Cumulative Mass Function (CMF) of the Poisson distribution to ask, “What is the chance of the error distribution producing the observed number of Johnny Darter sequences in a field sample?” For the Ac12s Johnny Darter detection, this results in Probability of (X>x) <0.0001. The conclusion is that for each field sample, the detection of Johnny Darter in Ac12s is unlikely due to contamination. But there are three additional markers. Applying the same approach, we find that for all samples across all markers there are sufficient Johnny Darter sequences in each sample to conclude the Johnny Darter detection is likely not due to contamination in any field sample (Appendix Table 1).

The same approach was applied to the remaining 15 species listed as detected in Juday Creek (Table S2). When we account for the possible detection due to contamination, there are 20 instances with some >0.001 possibility that contamination has led to the positive detection for a single species-marker combination. However, with the criteria that more than two sequences had to of been observed in at least two markers, this would only change our interpretation of three observations of the possible 128 (Appendix Table 2). The affected two species the Blue Gill and Yellow Bullhead. For the other species for which we observed a significant value (i.e., Rainbow Trout, Common Carp, and Brown Trout: see Table S10) there was enough evidence from the other markers that we could exclude the marker showing a possible contamination and still have enough sequence evidence to conclude its presence in the sample.

While three species-samples combinations changed from positive detection to no detection, all species are still detected and there is no change to the expected species richness. However, there is the potential that the contamination-corrected species counts for each field site have change the confidence intervals of the Chao estimators. There is very little difference between the Chao estimates and their 95% confidence intervals with and without contamination considered (Appendix Table 3). This is because there was no change in the total observed species richness (16) and the shift from detection to non-detection for the three samples (two samples for Yellow Bullhead and one sample for Bluegill) occurred for species where the sample incidence record shifted from eight to six and from eight to 7 for Yellow Bullhead and Bluegill respectively. Overall, we conclude that contamination has little influence on our conclusions about the observed or estimated species richness in Juday Creek.

**Appendix Table 1**: Parameter estimates (*λ*) and associated probabilities that the observed number of Johnny Darter sequences in field samples came from the distribution of errant DNA.

|  | | Juday Creek samples | | | | | | | |
| --- | --- | --- | --- | --- | --- | --- | --- | --- | --- |
|  |  | L1 | L2 | L3 | L4 | L5 | L6 | L7 | L8 |
| Marker | Ac12s  (*λ*=66) | <0.001 | <0.001 | <0.001 | <0.001 | <0.001 | <0.001 | <0.001 | <0.001 |
|  | Ac16s  (*λ*=6.5) | <0.001 | <0.001 | <0.001 | <0.001 | <0.001 | <0.001 | <0.001 | <0.001 |
|  | Am12s  (*λ*=12.25) | <0.001 | <0.001 | <0.001 | <0.001 | <0.001 | <0.001 | <0.001 | <0.001 |
|  | Cytb  (*λ*=92.5) | <0.001 | <0.001 | <0.001 | <0.001 | <0.001 | <0.001 | <0.001 | <0.001 |

**Appendix Table 2**: Detection of species as a result of presence of DNA in samples and consideration of contamination. Dark gray cells indicate species and samples that switched from detection to non-detection as a result of considering possible contamination leading to detection.

|  | **Sites in Juday Creek** | | | | | | | |
| --- | --- | --- | --- | --- | --- | --- | --- | --- |
|  | **Most downstream 🡨---------------------------------------------------------------------------🡪Most upstream** | | | | | | | |
| **Species** | **R1** | **R2** | **R3** | **R4** | **R5** | **R6** | **R7** | **R8** |
| Johnny Darter | Detection | Detection | Detection | Detection | Detection | Detection | Detection | Detection |
| Mottled Sculpin | Detection | Detection | Detection | Detection | Detection | Detection | Detection | Detection |
| White Sucker | Detection | Detection | Detection | Detection | Detection | Detection | Detection | Detection |
| Green Sunfish | Detection | Detection | Detection | Detection | Detection | Detection | Detection | Detection |
| Rainbow Trout | Detection | Detection | Detection | Detection | Detection | Detection | Detection | Detection |
| Creek Chub | Detection | Detection | Detection | Detection | Detection | Detection | Detection | Detection |
| Rock Bass | Detection | Detection | Detection | Detection | Detection | Detection | Detection | Detection |
| Bluegill | Non Detection | Detection | Detection | Detection | Detection | Detection | Detection | Detection |
| Largemouth Bass | Detection | Detection | Detection | Detection | Detection | Detection | Detection | Detection |
| Smallmouth Bass | Detection | Detection | Detection | Detection | Detection | Detection | Detection | Detection |
| Common Carp | Detection | Detection | Detection | Detection | Detection | Detection | Detection | Detection |
| Yellow Bullhead | Detection | Detection | Detection | Detection | Non Detection | Detection | Non Detection | Detection |
| Western Blacknose Dace | Detection | Non Detection | Non Detection | Non Detection | Detection | Detection | Detection | Detection |
| Rainbow Darter | Non Detection | Detection | Non Detection | Non Detection | Non Detection | Non Detection | Non Detection | Non Detection |
| Brown Trout | Detection | Detection | Detection | Detection | Non Detection | Non Detection | Non Detection | Non Detection |
| Eastern Mudminnow | Detection | Non Detection | Non Detection | Non Detection | Non Detection | Non Detection | Non Detection | Non Detection |

**Appendix Table 3:** Comparison of mean Chao estimator and confidence interval for incidence base accumulation curve without and with contamination considered.

| Sample | Without contamination considered  Mean (95% CI) | With contamination considered  Mean (95% CI) |
| --- | --- | --- |
| 1 | 13.74 | 13.31 |
| 2 | 14.51 | 14.49 |
| 3 | 14.95 | 14.92 |
| 4 | 15.33 | 15.33 |
| 5 | 15.58 (15.39, 20.17) | 15.58 (15.4, 20.19) |
| 6 | 16.12 (15.65, 23.60) | 16.12 (15.65, 23.61) |
| 7 | 16.62 (15.92, 26.86) | 16.62 (15.92, 26.87) |
| 8 | 16.88 (16.06, 28.65) | 16.88 (16.06, 28.65) |

**References**

McMurdie P. J. and Holmes S. (2014) Waste Not, Want Not: Why Rarefying Microbiome Data Is Inadmissible. *PLoS Computational Biology* 10(4): e1003531.

Murray DC, Coghlan ML, Bunce M. (2015) From benchtop to desktop: important considerations when designing amplicon sequencing workflows. *PloS ONE* 10:e0124671.

Valentini, A., Taberlet, P., Miaud, C., Civade, R., Herder, J., Thomsen, P. F., et al. (2015) Next-generation monitoring of aquatic biodiversity using environmental DNA metabarcoding. *Molecular Ecology*. Accepted Author Manuscript. doi:10.1111/mec.13428.
